# Gastric Cancer Staging: Is It Time for Magnetic Resonance Imaging?

**DOI:** 10.3390/cancers12061402

**Published:** 2020-05-29

**Authors:** Matteo Renzulli, Alfredo Clemente, Daniele Spinelli, Anna Maria Ierardi, Giovanni Marasco, Davide Farina, Stefano Brocchi, Matteo Ravaioli, Irene Pettinari, Matteo Cescon, Alfonso Reginelli, Salvatore Cappabianca, Gianpaolo Carrafiello, Rita Golfieri

**Affiliations:** 1 Radiology Unit, Department of Experimental, Diagnostic and Speciality Medicine, Sant’Orsola Hospital, University of Bologna, 40138 Bologna, Italy; daniele.nexus@gmail.com (D.S.); stefano.brocchi85@gmail.com (S.B.); irene.pettinari8725@gmail.com (I.P.); rita.golfieri@unibo.it (R.G.); 2 Radiology and Radiotherapy Unit, Department of Precision Medicine, University of Campania “L. Vanvitelli”, 80138 Naples, Italy; alf.clemente@hotmail.it (A.C.); alfonsoreginelli@hotmail.com (A.R.); salvatore.cappabianca@unicampania.it (S.C.); 3 Diagnostic and Interventional Radiology, ASST Santi Paolo e Carlo, San Paolo Hospital, 20142 Milan, Italy; amierardi@yahoo.it (A.M.I.); gcarraf@gmail.com (G.C.); 4 Department of Medical and Surgical Sciences, University of Bologna, 40138 Bologna, Italy; giovannimarasco89@gmail.com; 5 Department of Medical and Surgical Specialties, Radiological Sciences, and Public Health, University of Brescia, 25138 Brescia, Italy; davide.farina@unibs.it; 6 General and Transplant Surgery Unit, Department of Medical and Surgical Sciences, University of Bologna, 40138 Bologna, Italy; matteo.ravaioli@aosp.bo.it (M.R.); matteo.cescon@unibo.it (M.C.)

**Keywords:** gastric cancer, magnetic resonance imaging, treatment, diagnosis

## Abstract

Gastric cancer (GC) is a common cancer worldwide. Its incidence and mortality vary depending on geographic area, with the highest rates in Asian countries, particularly in China, Japan, and South Korea. Accurate imaging staging has become crucial for the application of various treatment strategies, especially for curative treatments in early stages. Unfortunately, most GCs are still diagnosed at an advanced stage, with the peritoneum (61–80%), distant lymph nodes (44–50%), and liver (26–38%) as the most common metastatic locations. Metastatic disease is limited to the peritoneum in 58% of cases; in nonperitoneal distant metastases, the most involved GC metastasization site is the liver (82%). The eighth edition of the tumor-node-metastasis staging system is the most commonly used system for determining GC prognosis. Endoscopic ultrasonography, computed tomography, and 18-fluorideoxyglucose positron emission tomography are historically the most accurate imaging techniques for GC staging. However, studies have recently shown renewed interest in magnetic resonance imaging (MRI) as a useful tool in GC staging, especially for distant metastasis assessment. The technical improvement of diffusion-weighted imaging and the increasing use of hepatobiliary contrast agents have been shown to increase the diagnostic performance of MRI, particularly for detecting peritoneal and liver metastasis. However, no principal oncological guidelines have included the use of MRI as a first-line technique for distant metastasis evaluation during the GC staging process, such as the National Comprehensive Cancer Network Guidelines. This review analyzed the role of the principal imaging techniques in GC diagnosis and staging, focusing on the potential role of MRI, especially for assessing peritoneal and liver metastases.

## 1. Introduction

Gastric cancer (GC) is one of the most common malignancies of the digestive system [1] and is the fifth most common tumor among overall cancers, with an incidence of one million new patients diagnosed every year [2,3]. Fortunately, in the 21st century, improvements in worldwide sanitary conditions, diet, and medical advances have effected a significant reduction in the incidence of GC, especially in those patients with advanced disease, usually called advanced GC [2]. However, this malignant tumor still remains a significant cause of morbidity and mortality worldwide (fifth most common malignancy in the world) due to its global diffusion, associated with common risk factors such as diet, *Helicobacter pylori*, tobacco, obesity, adenomatous polyps, chronic atrophic gastritis, and the male sex [4].

The incidence of GC varies greatly depending on geographic area, which is similar to what has been observed in other tumors: it remains the first cause of mortality from malignant cancers in Eastern Asia, and nearly two-thirds of global GCs occur in developing areas. The highest incidence occurs in Japan and South Korea, and the most involved population is the Chinese, who represent almost 43% of all cases. This figure appears to be correlated with particular diets and hygienic conditions [5].

Unfortunately, GC is characterized by a high mortality rate: the five-year overall mortality is 70–80%. At present, the best survival rate is in the Japanese (52% five-year survival), probably due to the effective screening programs for GC that began in the early 1990s [6,7,8].

The term “gastric cancer” is commonly used to refer to adenocarcinoma, which represents between 90% and 95% of all gastric malignancies. However, benign lesions commonly comprise approximately 85–90% of all lesions found within the stomach [8]. There are multiple subtypes of GC, including papillary, tubular, and signed ring cell forms. This high variability in histotypes corresponds to the varying appearance of adenocarcinoma on imaging. In fact, it could appear as a bulky mass, sometimes with ulceration, such as a gastric wall thickening or a diffuse parietal infiltration (without a visible lesion), or as a particular presentation called “linitis plastica” [9]. Moreover, one distinctive but uncommon appearance of GC is that of mucinous adenocarcinoma, which can partially calcify [10]. Accurate tumor-node-metastasis (TNM) staging of GC is the cornerstone (central pin) of prognostication in GC and for performing the most accurate decision-making for treatment, reducing unnecessary surgery, and maximizing the likelihood of benefiting from selected treatments [1,11].

The imaging techniques typically performed for the diagnosis of GC and for performing its staging are endoscopic ultrasonography (EUS), computed tomography (CT), and positron emission tomography (PET)-CT [3,12,13]. Historically, magnetic resonance imaging (MRI) had a limited role in GC evaluation due to its technical limitations, such as blurring and lower spatial resolution. In recent decades, however, much progress has been made in MRI technology, improving the diagnostic performance of this imaging technique in many areas of medicine, including oncology [14]. Furthermore, MRI presents many advantages over CT (the radiological method recommended for the staging of GC), including the ability to generate significantly greater soft-tissue contrast resolution and the ability to remove the risk of iodinated contrast-induced nephropathy or ionizing radiation [15]. However, in the guidelines for the management of GC, MRI does not appear among the possible imaging techniques useful for GC staging [12,13].

This study aimed to analyze the role of the imaging techniques used in the diagnosis and staging of GC, focusing on the potential role of MRI, especially in the assessment of liver and peritoneal metastases from GC.

## 2. Tumor-Node-Metastasis Staging of Gastric Cancer 

The pivotal role of correct GC imaging lies in the possibility of performing different treatments in various stages of disease, especially curative treatments in the early stages. For example, selected patients with early GC could be treated with endoscopic resection, especially if the lesion is easily approachable during the endoscopic examination [16,17]. In patients with a locally advanced disease but without distant metastasis, perioperative chemotherapy, (sub)total gastrectomy, and regional lymph node dissection are the current acceptable standard of care [16,17]. The poorest clinical scenario is metastatic disease. Patients with a solitary liver metastasis can be treated with surgical resection. However, it is generally accepted that patients with GC at stage M1 are incurable and that they should be managed with noncurative intent [16,17,18,19]. The TNM guidelines recommend the use of EUS to accurately evaluate the extension of the primary tumor (T parameter) [3,12,13]. This technique is sufficient to diagnose the GC stage until T3 (tumor penetrates subserosal connective tissue without invasion of visceral peritoneum or adjacent structures) [3,12,13]. In fact, EUS has demonstrated high diagnostic performance in distinguishing the different layers that compose the gastric wall, visualize the perigastric lymph nodes and detect intraperitoneal fluid utilizing of a modern miniaturized US probe [20]. Conversely, CT has insufficient spatial resolution and soft-tissue contrast resolution to distinguish the layers of the gastric wall, and therefore it is not accurate for evaluating T1, T2, or T3 parameters of GC. However, CT is useful for identifying extragastric invasion (T4) [12,13]. Therefore, for stage T4, the use of CT and then PET-CT is still recommended to assess tumor nodularity outside the stomach or invasion of adjacent organs [12,13]. Finally, MRI is not recommended for the imaging evaluation of the T parameter in GC (Table 1) [13].

According to the guidelines for the management of GC, the N parameter (lymph node involvement) is assessed by using CT or PET-CT [12,13]. In particular, potential lymph node metastasis is suspected (1) when the short axis measures greater than 1 cm on CT of the abdomen or on PET-CT and (2) when fluorodeoxyglucose-avid lymph nodes are detected on PET-CT. The criterion for differentiation between stages N1 through N3 is represented exclusively by the number of involved lymph nodes: metastases in one to two regional lymph nodes corresponds to N1, three to six correspond to N2, and seven or more correspond to N3 (N3a, metastasis in 7–15 regional lymph nodes; N3b, metastasis in 16 or more regional lymph nodes). Lymph node count should be assessed using CT or PET-CT. Moreover, pathologically involved lymph nodes inferior to the level of the renal veins are treated as systemic metastases for the purposes of staging [12,13]. Finally, MRI is not recommended for the imaging evaluation of the N parameter in GC (Table 1) [13].

Until 2017, it was recommended that the M parameter (metastatic disease) be evaluated by CT or PET-CT and by the N parameter. Therefore, MRI is not recommended for the imaging evaluation of the M parameter in GC (Table 1) [13].

Is there robust scientific evidence to support the current absence of MRI in the guidelines for the management of GC?

## 3. Magnetic Resonance Imaging in T- and N-Parameter Evaluation of Gastric Cancer

A meta-analysis, published by Seevaratnam et al. [21] in 2012, compared the diagnostic performance of the most common imaging techniques used for the staging of GC—EUS, CT, MRI, and PET. This study, which included a total of 40 articles from 1 January 1998 to 1 December 2009, for a total of 3758 patients, clearly conveyed the mistaken belief that CT is more reliable than MRI for TNM staging of GC. The authors demonstrated that the overall accuracy in the assessment of T stages of MRI (82.9% ± 3.7%) was statistically superior to that of CT (71.5% ± 2.7%) (*p* ≤ 0.014). MRI also appeared to be better than CT in terms of sensitivity in assessing the N parameter of GC, with values of 85.3% and 77.2%, respectively. However, this study presented an important limitation: the authors did not consider the role of MRI for the diagnosis of metastasis (M parameter) because they considered MRI as “limited” due to the insufficient amount of body evaluable within a single examination and as thus unsuitable for staging the M parameter [21]. In their conclusion, however, Seevaratnamm et al. [21] declared the superiority of MRI compared with CT, despite the number of MRI studies analyzed being fewer than the CT studies.

Subsequently, in 2015, a systematic review and meta-analysis conducted by Huang et al. [22] evaluated the utility of MRI in preoperative T and N staging of GC. Eleven studies were included in the analysis, and the final results revealed surprising data concerning the diagnostic performance of MRI in this setting. In fact, the reported pooled sensitivity of MRI in diagnosing the T1, T2, T3, and T4 stages of GC was 66%, 85%, 86%, and 88%, respectively, and the pooled specificity for the same stages was 97%, 90%, 89%, and 97%, respectively. Considering T3 and T4 together, the pooled sensitivity and specificity were 93% and 91%, respectively. Moreover, this study confirmed the remarkable pooled sensitivity (86%) of MRI for the correct assessment of the N parameter, despite a loss in specificity, confirming the data reported in other studies [23,24]. In a subgroup analysis, the authors [22] tested the diagnostic utility of diffusion-weighted imaging (DWI) plus MRI compared with MRI alone. Although DWI appears to be superior for the detection of lesions from GC, no statistical added value with respect to MRI alone emerged from their analysis. These early results have been partially disavowed by subsequent studies that assessed the usefulness of DWI in this setting.

In a study by Soydan et al. [25] performed on a total of 46 patients who underwent abdominal DWI-MRI before surgery for GC, the sensitivity, specificity, and accuracy of DWI-MRI in T-staging produced satisfying results—in particular, that DWI-MRI could distinguish T4 from lower stages of GC with great reliability. In fact, the authors concluded that DWI-MRI could consistently help to determine an accurate staging of nonmetastatic GC. This thesis is also supported by other studies in which DWI also demonstrated better performance due to greater soft-tissue resolution than CT, reducing artifacts and with the advantage of revealing more details of GC (depicting characteristics such as necrotic, cystic, or bleeding aspects) [23,24].

Joo et al. evaluated the diagnostic performance of MRI performed alone and in combination with DWI for the preoperative TNM staging of GC, especially for assessing metastatic lymph nodes. The MRI performed using DWI had demonstrated a higher sensitivity than MRI without DWI for N staging (86.7% vs. 50%) but a lower specificity (58.8% vs. 90%) [26]. Moreover, the alteration of cellular density, evaluable in metastasis using DWI, has further increased its potentiality, especially in cases of subcentimetric infiltrated lymph nodes [27].

Unfortunately, none of these authors considered studies in which the possibility of using MRI as a “one stop” imaging technique to diagnose any TNM parameters during the preoperative phase was evaluated. It is difficult to determine the reasons for the failure to develop MRI for GC staging.

## 4. Magnetic Resonance Imaging of Metastases from Gastric Cancer

A substantial portion of patients with newly diagnosed GC have distant metastases (M1 disease). Stage IV disease, consisting of any T or N and M1, the most advanced form of GC, is diagnosed in 35–55% of GC cases in low-incidence countries, such as the United States and Canada [28,29]. The median disease-specific survival in metastatic disease has been estimated to be approximately 10 months, and overall 5-year survival is estimated to be 3–5% [11,29,30]. Finally, it is important to perform a correct diagnosis of distant metastases in GC, because it dramatically changes prognosis and treatment plans [31].

The most common sites of distant metastases from GC are the peritoneum in 61–80% of cases, the distant lymph nodes in 44–50%, and the liver in 26–38%. Following these sites, there are few minor locations for secondary malignancies from GC, such as the lung (10%), bone (6%), abdominal wall (2%), ovary (2%), brain, and prostate (approximately 0.4% for both) [32,33]. Metastatic disease was limited to the peritoneum in 58% of M1 patients, whereas 20% of M1 disease showed exclusively nonperitoneal distant metastases. In this latter case, the most involved GC metastasis site was the liver, with a weighty value of 82%, much more than the two most affected sites after the liver, lung, and bone, both equally involved in approximately 9% of cases [34].

Our study aimed to highlight the emerging role of MRI in the detection and characterization of the most common metastases from GC, such as peritoneal and liver metastases. Furthermore, this study aimed to analyze whether there are robust scientific data to explain why MRI was not introduced in the guidelines as a useful tool in the staging of patients affected by GC, given scientific data on its diagnostic accuracy in the assessment of peritoneal and liver metastases from GC are robust.

## 5. MRI in the Assessment of Peritoneal Metastases from Gastric Cancer

A systematic review by Wang et al. [35], comparing CT and MRI in the assessment of hepatic and peritoneal metastases from GC, demonstrated that CT showed high sensitivity and specificity in evaluating peritoneal and liver metastases from GC. However, MRI results also appeared to be accurate, with a diagnostic performance comparable with that of CT, potentially even better. MRI was described as a growing imaging method for the detection and characterization of most diffused repetitions from the oncologic pathologies of the abdomen, including GC. In fact, MRI demonstrated high sensitivity and specificity in detecting liver metastasis, with a sensitivity of 100% (95% Confidence Interval (CI) 0.40–1.00) and specificity of 100% (95% CI 0.89–1.00). Furthermore, DWI-MRI was more sensitive than CT in detecting liver and peritoneal metastases, and MRI functional parameters such as apparent diffusion coefficient were found to help evaluate the pathological response to neoadjuvant chemotherapy [36].

Therefore, over 10 years ago, there was already the strong suspicion that MRI could be a promising method for also evaluating peritoneal metastasis. Unfortunately, in 2011, the lack of data in this diagnostic field (MRI in the detection of peritoneal metastasis) did not facilitate the demonstration of MRI reliability in this oncological setting compared with other available imaging techniques. In fact, among 33 overall studies included in the review [35], only two MRI-based papers were included along with 22 CT studies. However, as early as 2011, these first results made it possible to define MRI as a “promising technology” that could represent the future of the imaging of metastases.

Similarly, a systematic review published by Laghi et al. [37] in 2017 assessed the diagnostic accuracy of CT and MRI in detecting peritoneal metastases. The authors analyzed a total of 22 studies, of which only three were MRI-based, whereas 19 were CT-based. The overall final population consisted of 630 patients, of which 275 were reported metastases from gastrointestinal malignancies. In this study, both MRI specificity and sensitivity were greater than those of CT, although without statistical significance. In particular, MRI sensitivity and specificity were 86% (95% CI 0.78–0.93) and 88% (95% CI 0.83–0.92), respectively, versus CT sensitivity and specificity of 83% (95% CI 0.79–0.86) and 86% (95% CI 0.82–0.89), respectively. However, the main limitation of this study, as in that of Wang [35], was the small sample size of the MRI population, which did not allow us to draw definitive conclusions on this imaging modality. In fact, the authors concluded that the MRI technique represents a future resource more than a current reality.

In recent years, an increasing number of scientific papers have stressed the need for a correct preoperative diagnosis of distant metastases from GC, particularly peritoneal metastases, to reduce need for a second imaging technique to achieve the final diagnosis and, most of all, to prevent unnecessary surgical treatments. In this context, Borggreve et al. [1] reported that, although MRI is rarely considered for the diagnosis of peritoneal metastasis, this technique demonstrated the best sensitivity, specificity, and diagnostic accuracy for this type of diagnosis compared with other imaging modalities. In particular, the accuracy, sensitivity, and specificity for detection of peritoneal metastases of PET-CT and DWI-MRI were 80%, 84%, and 73% and 83%, 84%, and 82%, respectively.

Another recent interesting study, comparing MRI and CT in terms of accuracy for detecting peritoneal ovarian metastases, demonstrated the clear superiority of MRI (93.33%) over CT (79.39%) and even further highlighted the lower “omission rate” of MRI. In fact, the authors observed that MRI missed the detection of only 12 metastatic lesions (6.67%) compared with CT, which omitted 34 lesions (20.61%), in 165 overall secondary malignancies [38]. This poor performance of CT was also confirmed by another study, in which the omission rate for diagnosis of peritoneal metastasis from GC was approximately 16%. In fact, the authors proposed a steep learning curve to improve CT performance; however, it will be some time before it is affordable and available in clinical practice. 

Current guidelines recommend staging laparoscopy (SL) for advanced GC [13]. Despite many developments in preoperative imaging, the suboptimal identification of advanced carcinomatosis disease could result in high rates of unnecessary laparotomies in patients with GC [39]. The addition of peritoneal lavage can identify patients with free intraperitoneal cancer cells, which suggests a poor prognosis. Although considered as a very useful asset in the preoperative cancer staging, the wider application of SL is been long debated [39]. Awaiting further robust data concerning non-invasive imaging techniques such as MRI in the management of GC, the goal of MRI could be to achieve high sensitivity in detecting peritoneal metastases to reduce the false negative cases who undergo unnecessary surgical procedures. This as a primary result could allow reducing any invasive and mini-invasive procedures, improving patient quality of life. Therefore, according to the confirmed results emerging from the literature, MRI is actually ready to become the “first choice” imaging in the evaluation of peritoneal metastasis, also from GC [40].

## 6. Magnetic Resonance Imaging in the Assessment of Liver Metastases from Gastric Cancer

The liver is by far the most common site of distant metastases in patients with nonperitoneal secondary lesions from GC. A recent review and meta-analysis had compared the diagnostic accuracy of MRI performed with hepatospecific contrast media, such as gadolinium ethoxybenzyl diethylenetriamine pentaacetic acid (Gd-EOB-DTPA) with that of contrast-enhanced CT (CE-CT) in patients with liver metastases [41]. It was demonstrated that the sensitivity of Gd-EOB-DTPA MRI was significantly higher than that of CE-CT. More precisely, this study investigated the diagnostic potential and precision of these imaging techniques on patients with only liver metastases from gastrointestinal and colorectal primitive cancers. The study performed by Australian Safety and Efficacy Register of New Interventional Procedures Surgical included nine diagnostic accuracy studies from 1991 to 2016, reassembling a total sample size population of 537 patients with a total of 1216 lesions. The median per-lesion sensitivity of Gd-EOB-DTPA MRI was 94.9% (range, 86.9–100.0%), and that of CE-CT was 74.2% (range, 51.8–84.6%), with statistical differences in favor of MRI (*p* < 0.001). Interestingly, this statistically significant advantage of Gd-EOB-DTPA MRI over CT in terms of sensitivity in detection of liver metastases does not make MRI poorer in terms of specificity (median 86.6% (range, 77.2–98%) for MRI and 94.1% (range, 80.2–98%) for CT; *p* = 0.44). A further per-lesion subgroup analysis for metastases other than those from colorectal cancers showed that Gd-EOB-DTPA MRI was 1.36 times more sensitive than CE-CT (95% CI 1.13–1.65; *p* = 0.001).

Moreover, the authors [41] analyzed the imaging sensitivity according to the lesion’s diameter, dividing the patient population into two main groups: lesions <10 mm and lesions ≥10 mm. For lesions smaller than 10 mm, the median per-lesion sensitivity of Gd-EOB-DTPA MRI was 85.7% (range, 80.7–92.3%), and that of CE-CT was 50.0% (range, 26.0–64.5%). Therefore, the sensitivity of Gd-EOB-DTPA MRI appeared statistically superior to that of CE-CT (*p* < 0.001). As expected, these results were not confirmed for lesions larger than 10 mm, for which the median sensitivity of Gd-EOB-DTPA MRI (96.9%) remained higher than that of CE-CT (92.9%), but without statistical significance. These data confirm that Gd-EOB-DTPA MRI is more sensitive than CE-CT for detecting all liver metastases, but particularly those smaller than 10 mm [41].

A milestone study in the diagnosis of liver metastases has been shown in a meta-analysis published in 2016 by Vilgrain et al. [42]. This study assessed the global diagnostic performance of DWI alone, Gd-EOB-DTPA MRI alone, and the combination of both techniques in the detection of liver metastases. The study included 39 articles, with a total final population of 1989 patients and 3854 overall lesions. The per-lesion sensitivities of DWI alone, Gd-EOB-DTPA MRI alone, and the combined techniques were 95.5%, 90.6%, and 87.1%, respectively, with a *p*-value < 0.001, comparing the combined technique with the other techniques alone, and Gd-EOB-DTPA MRI alone with DWI alone. These data were subsequently confirmed by other studies that analyzed these three MRI techniques [14,43]. The resultant high sensitivity of MRI justifies the use of this technique in all patients with liver metastases to ensure the most accurate preoperative planning [14,43].

**Figure 1:**
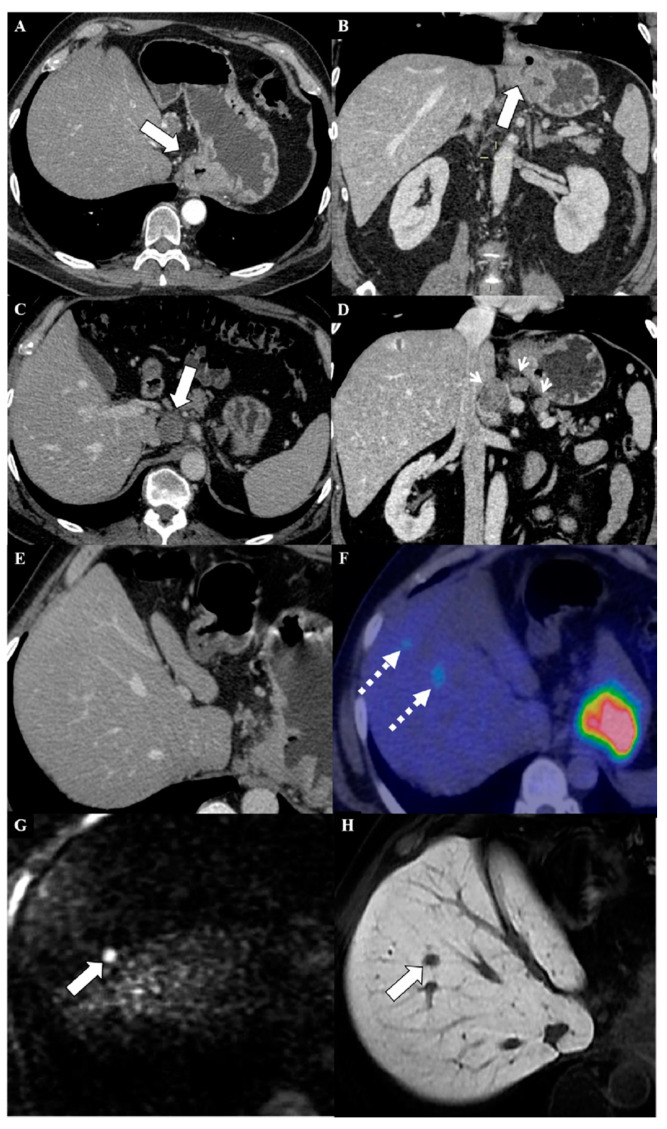
A 62-year-old man complaining of dysphagia and epigastric pain with advanced stage gastric cancer. Axial (**A**) and coronal reformatted (**B**) contrast-enhanced computed tomography (CT) images showed a circumferential soft-tissue thickening involving the gastroesophageal junction and the cardia of the stomach (white arrows) invading the serosa layer (T-stage: T4a). Multiple (3) enlarged lymph nodes were appreciable in the aortocaval (**C**) (white arrow) and celiac (**D**) (arrowheads) region (N-stage: N2). Axial contrast-enhanced CT scan (**E**) did not demonstrate liver metastasis, whereas the axial ^18^FDG positron emission tomography computed tomography (PET-CT) image (**F**) revealed two metabolically active lesions with the same uptake value (SUVmax 4.7) located in the VIII hepatic segment (dotted arrows), consistent with M1 disease. The subsequent axial gadoxetic acid-enhanced magnetic resonance imaging, performed using diffusion-weighted imaging (**G**) and during the hepatobiliary phase (**H**), confirmed only one metastatic lesion (white arrow).

**Figure 2:**
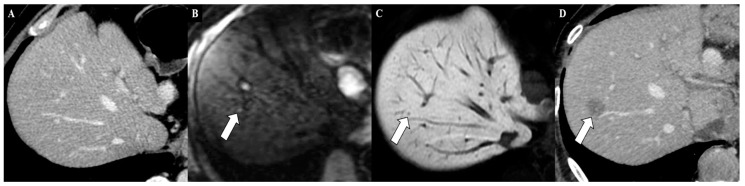
Axial contrast-enhanced computed tomography (CT) image (**A**), in the same patient, did not show metastatic lesions at this liver level. The axial gadoxetic acid-enhanced magnetic resonance imaging, performed using diffusion-weighted imaging (**B**) and during the hepatobiliary phase **(C)**, demonstrated a small liver metastasis in the liver segment VIII (white arrows). After six months, the axial contrast-enhanced CT image (**D**) confirmed the metastatic liver lesion (white arrow), enlarged.

Moreover, another reason for the implementation of MRI and for its use in the staging of patients with cancers, including GC, also lies in the epidemiology of benign liver lesions. Benign hepatic tumors and tumor-like conditions have been demonstrated to occur in 52% of men with these lesions, and this rate is considerable because it does not include liver cysts [44]. Moreover, the number of benign lesions increases with the mean patient age [44]. This finding, discovered many years ago, is accurate; various studies about treating fortuitously discovered liver lesions have confirmed this high rate of benign lesions in the general population [45]. Therefore, it is mandatory to determine the correct stage for an oncological patient, to use an imaging technique with high diagnostic performance in detecting metastasis, and also to be able to correctly characterize hepatic lesions in both noncirrhotic and cirrhotic livers [46,47,48]. In the near future, if the data are confirmed, MRI could become the imaging of choice in the staging of oncological patients, including patients with GC (Figure 1 and Figure 2).

## 7. Conclusions

MRI has been demonstrated to be an accurate technique for the staging of GC. In particular, the available results concerning the diagnostic performance of MRI in detecting peritoneal metastases from GC are promising, although the data are still limited. Further comparative studies between MRI and other imaging modalities for GC staging are needed in order to assess the possible added value of MRI in the management of patients affected by GC. From the point of view of liver metastases from GC, many studies have shown robust evidence that MRI with hepatospecific contrast media and with DWI currently represent the most accurate imaging technique. According to the confirmed results emerging from the literature, MRI is ready to become the “first choice” imaging in the staging of GC.

## Figures and Tables

**Table 1:** Eighth edition American Joint Committee on Cancer (AJCC) Tumor Nodes Metastasis (TNM) staging for gastric neoplasms with suggested imaging for staging (adapted from Amin et al. [12] and from the National Comprehensive Cancer Network (NCCN) Guidelines [13]).

8th AJCC Gastric Cancer TNM Staging	Definition	Imaging
**T**		
TX	Primary tumor cannot be assessed	EUS is the modality of choice in assessing the invasion depth of gastric cancer.
T0	No evidence of primary tumor	
Tis	Carcinoma in situ: intraepithelial tumor without invasion of the lamina propria	
T1	Tumor invades lamina propria, muscularis mucosae, or submucosa	
T1a	Tumor invades lamina propria or muscularis mucosae	
T1b	Tumor invades submucosa	
T2	Tumor invades muscularis propria	
T3	Tumor penetrates subserosal connective tissue without invasion of visceral peritoneum or adjacent structures T3 tumors also include those extending into the gastrocolic or gastrohepatic ligaments or into the greater or lesser omentum, without perforation of the visceral peritoneum covering these structures	
T4	Tumor invades serosa (visceral peritoneum) or adjacent structures	CT is more useful in advanced T stages to evaluate the tumor nodularity outside
T4a	Tumor invades serosa (visceral peritoneum)	the stomach or invasion of the adjacent organs.
T4b	Tumor invades adjacent structures, such as the spleen, transverse colon, liver, diaphragm, pancreas, abdominal wall, adrenal gland, kidney, small intestine, and retroperitoneum	
**N**		
NX	Regional lymph node(s) cannot be assessed	CT, PET/CT, and EUS can be used for nodal staging. Radiological criterion for nodal involvement is lymph node enlargement.
N0	No regional lymph node metastasis	
N1	Metastasis in 1 to 2 regional lymph nodes	
N2	Metastasis in 3 to 6 regional lymph nodes	
N3	Metastasis in 7 or more regional lymph nodes	
N3a	Metastasis in 7–15 regional lymph nodes	
N3b	Metastasis in 16 or more regional lymph nodes	
**M**		
M0	No distant metastasis	CT or PET/CT are recommended to assess metastatic disease.
M1	Distant metastasis	

CT, computed tomography; MRI, magnetic resonance imaging; PET/CT, 18F-fluorodeoxyglucose positron emission tomography; EUS, endoscopic ultrasound.
